# Chicago Medical Society

**Published:** 1888-06

**Authors:** 


STATED MEETING, APRIL 16.

President J. H. Etheridge in the chair.

Professor L. L. McArthur read a paper entitled "A New Abuse of Hospital Charity," in which he said: As I understand it, the aim of this society is not confined solely to the consideration of questions of scientific interest, but is to exercise also a sort of guardianship over the interests and welfare of the medical profession in general. I select this means therefore of calling the attention of the medical men of this country—for it is a question of general, not local, interest—because it may thus be better brought to their attention. "What's everybody's business is nobody's," is a trite saying, but applicable here, and unless some one takes the initiative, nothing will be accomplished; so, feeling as I do, that the interest of one is the interest of all, permit me to call your attention to a new abuse of the physician's charitable work. During the past few years there have been organized through the United States several alleged chartered corporations, styling themselves National Benefit Associations, Mutual Hospital Aid Associations, and the like, and offering for a small pecuniary consideration ($6 to $10) to insure *for one year* the provision of hospital care, medical service, nursing, medicines, etc., as follows:

A circular of one of them is as folllows:

The object and business of this company are to provide the best possible care and treatment for men wherever they may happen to be when sick or hurt, in consideratiun of a comparatively nominal annual payment.

To accomplish this, arrangements have been made with a large number of the best hospitals in the country (see list herewith), by which any man presenting one of our certificates will be admitted, and kindly cared for, and skillfully treated without further expense to himself.

A large class of men in this country who are earning their living in the mills, and foundries, and large manufactories, or on railroads, in the logging camps, and mines, and the thousand industries that are developed by their labor, are subject constantly to severe sickness or injury, and generally find themselves at such a time sadly in need of the comforts, nursing, and proper food necessary to the rapid recovery of their health, and the healing of their wounds.

They are all right as long as they are strong and well, but when sickness or severe injury prostrates them, the strongest and roughest of them are like the merest child, and long for the comforts of home and the tender touch and intelligent care of some one who sympathizes with them in their sufferings. The place for them *then* is at a first-class hospital, where every provision is made for their care. Neat and quiet rooms, nurses trained and devoted to their high duty, skillful surgeons and physicians, medicine and apparatus in all forms known to the profession, and food carefully prepared for each patient's special case.

Our plan places all these necessities and comforts within the reach of the poorest man. With one of our certificates in his pocket, no matter where he is, he can rest assured that, if anything should happen to him, a hospital near by will welcome him, not as an object of charity, but as entitled to all its privileges, for he has paid for them in advance.

Hundreds of our certificate holders who have been in the hospitals from time to time during the past year, are ready to testify to the value of their investment, which amounts to only three cents a day, and are now most enthusiastic in praise of our plan.

Our certificates are sold by traveling agents, and at the hospitals, and will be sent by mail from our office. Each has the name of the chain of hospitals printed upon it, so that the holder may know where to apply, if occasion require, in any part of the country.

By the above you will see that no matter how well able to pay his physician a man may be, by the payment of $10 he is insured medical services for a period of one year, with or without the physician being consulted in the case. Upon investigation, however, these insurance companies turn out to be "one man" institutions, for whose sole benefit the scheme is devised and without his further exertion than to care for the cash account.

Their methods of obtaining business have been particularly shrewd and catching, something after this manner: The agent—*i. e.*, the company—goes or writes to every hospital of which he can obtain the address, states that he represents some influential insurance association, and in case of any of its members desirous to know the rates at which he may send them to that hospital. Having been told the usual price, he manages to make a contract with the hospital authorities to send all their cases for that district to that institution as per agreement. At first sight, to the hospital authorities, it seems a perfectly fair and just agreement, and they so sign for a number of years, thinking to lose nothing by taking them at cost price, and perhaps gain something by the advertising. A moment's consideration, however, will show that this is an imposition on the physician, who, however ready he may be to do a true charity, ought, if not in his own, at least in his fellows' interest, to emphatically object. We have already sufficient abuse of charity without permitting another scheme for the benefit of one at the expense of the many to be added to those existing. Of the long list of the most prominent hospitals in this country some have already perceived the injustice, and refused to extend their contracts, and were it brought by the staffs of the respective hospitals to the attention of the proper authorities I have no doubt they would refuse to renew those contracts already existing. In conclusion, let me say that no personal animosities or considerations have induced the presentation of this paper, but simply a desire to prevent the further growth of an already prospering imposition.

This institution in Chicago, of which the circular is being passed around, is run in elegant style in the Phœnix building, with a large suite of offices. A man takes the $10 and insures the other man that he shall have a doctor's care the year round, if necessary, and the doctor receives no compensation for it, though many times the man is perfectly able to pay for it. He goes to a railroad president or vice-president and says, I have this long list of hospitals in my contract and I would like you to recommend it to your employés as a very cheap form of hospital insurance. He sees that it is a good thing for the men, and does so. The "one man" makes an easy living at the expense of the hospital surgeons and physicians, who must turn out at all hours of the day or night to benefit him.

Dr. C. S. Bacon: From the description of the association given by the doctor, it seems that this is only one phase of the insurance business, and I do not understand that the companies fail to live up to their agreement. As far as appears from the paper they will in every case provide the hospital attendance, and as long as the company is a legal one, I do not see how it is to be blamed. If there is any blame it must attach to the hospital which will make such an agreement, and the paper is valuable as calling attention to the fact that hospitals, in this matter as in many other cases, are not just to the staff of attending physicians and surgeons. This is not a solitary case. It is well known that in many of the hospitals of this city, as in other places, patients are admitted who are well able to pay the attending physician, but by the rules of the hospital the physician must give his services for nothing. It is only by counting on the gratuitous services of the physician that they make the contract with the association. The blame, I should think, was entirely with the hospital and not with the association, which is a legally organized and honorably conducted insurance association.

Dr. George W. Webster: I would just remark that a man came to see me a few days ago, from Iowa, who had lost one of his hands in an accident. He said he had one of these certificates from an insurance company—he had been insured in this city. He said he presented that at two hospitals in the State in which he was injured, two hospitals that were on the printed list of the company in which he was insured, and both hospitals denied having made any agreement of that kind, and he was not allowed to enter either of them. He said the company was simply a fraud.

Professor McArthur, in closing the discussion, said: In regard to the location of the blame; I do not blame the man for trying to make as much out of it as he can. He does not do it for love, and the only ones who suffer are the physicians. In those cases in which the matter has been properly presented to the hospitals, for instance, St. Luke's, the trustees of the hospitals have refused to renew these contracts; I am pleased to know that they have done this in Iowa.

The President: I would like to ask the doctor if he knows the rate charged by these hospitals?

Professor McArthur: Six dollars a week for ward, and $10 for private room.

The President: Do they make a special contract or reduction from these rates?

Professor McArthur: The hospitals let them in on the ground floor and will take them at $6 a week and make a contract with the company, but the company make a contract with the insured for $6 or $10.

The President: I want to find out where this institution makes the money. The ordinary charge is $6 in the hospitals, and the hospitals must make a special rate lower than that in order for the association to make money.

Professor McArthur: The hospitals charge them at the rate which is figured at the end of the year as the average cost per patient, not to make, not to lose, $1.03 for St. Luke's. The money is made by the company by dealing in undelivered futures. They insure hospital care and medical services which in the majority of cases is never delivered. When delivered, no expense to company for medical care, no matter how long or how valuable. That is upon the annual cost, and they charge them the same rate. This institution has been going for a year, one in Portland, Oregon, for two years. One started up in Detroit, and one in Louisville.

Dr. R. TILLEY : Are all these associations run by the same person?

Professor MCARTHUR: No, sir; by different men. The Chicago association has a single representative.

Dr. Edwin Pynchon read a paper on the "Trituration of Alkaloids." He said: In its progress the science of medicine receives much assistance from the collateral professions, among which pharmacy and chemistry have been most liberal contributors. Of the products of chemistry the alkaloids merit consideration as being among the first in importance. In the alkaloid of a plant we have in a concentrated and invariable form the value—the medicinal worth of the plant. I say invariably, as a given alkaioid has ever the same chemical formula. In several cases a drug has been found to contain two or more alkaloids, though in such cases one of the same possesses pre-eminently, as compared with the others, those powers or properties which have been attributed to the crude drug. I believe where but one alkaloid is found it possesses therapeutically the properties of the mother remedy.

Two specimens of the same plant may differ as to the per cent. of alkaloid which they will yield. In other words, pound for pound, they differ in richness as to the quantity each may respectively contain of their active principle. In such case should a fluid extract be made from each specimen in accordance with the directions of the pharmacopœia how could it be expected that the two extracts would be of uniform strength? This condition of variance has been taken note of by some of our leading manufacturing pharmacists, who, in case of a few of the more active remedies, have attached to the container a note giving the per cent. of alkaloid which analysis has proven the preparation to contain. One manufacturer goes further by giving with each of the fluid preparations which he prepares, representing pound for pound, a printed formula for a test to prove that the preparation contains a required per cent. of the active principle. By a judicious mixing of the products of different percolations or distillations, as well as by dilution, a standard, which, though arbitrary, can be retained. In this way an uniformity of preparation is secured which cannot be had by following the pharmacopœia and is undoubtedly a step in the right direction.

If then, for example, the physician prescribes a fluid extract in order to obtain the effects of the alkaloid which it contains, why not prescribe the alkaloid itself and omit the starchy, coloring, and other inert matter of which the extract is composed? This query is equally applicable in case of the solid extract, tincture, etc. The more general employment of alkaloids has been subject to several objections. In but comparatively few drug stores can a full line of alkaloids be found. Again, as compared with more crude preparations, they are expensive, the greater number of them being imported, and principally of German manufacture. Other objections to their employment are the minuteness of the dose, requiring more delicate prescription scales than the average drug store is provided with, and particularly the difficulty in always remembering the *exact* dose, the importance of which, owing to the activity of the remedy, will suggest itself. The object of this paper is to show a means whereby these latter objections can be overcome.

In city practice, where drug stores are accessible, it is customary to write prescriptions, but in many places and cases, were it necessary to await the arrival from the drug store of the remedies prescribed, the case would either have recovered or have terminated fatally. I favor the use of the pocket medicine case, containing a reasonable number of appropriate remedies with which to meet the most common indications found in acute diseases and emergency cases. By the use of the medicine case and through the palatableness of the remedies therein contained, the homœopathists have been enabled to secure much practice which would otherwise have fallen into the hands of regular practitioners. The employment or the pocket medicine case, will, to a certain extent, do away with the annoyance and danger of the substitution and adulteration which are occasionally practiced by disreputable pharmacists in the compounding of prescriptions, for no physician for his own use would buy other than the best and purest which can be obtained, and as the profit on compounding prescriptions is generally large, the physician can frequently furnish one or more of the needed remedies at one-tenth of the cost which the patient would entail at the corner drug store. This financial side of the question has often been the cause of the patient employing one of that school of medicine the practitioners of which furnish their remedies without charge. And again, when prescriptions are invariably written it often occurs that the druggist gets all the money which the patient can spare, and the poor *medicus* waits, and continues waiting for his pay until the patient's gratitude has been effaced by time, and while thus waiting not infrequently learns that death has come to cancel the obligation; whereas, had he furnished a portion of the medicine required he might have received at least a portion of his fees.

When, in addition to the foregoing, we take into consideration the promptness with which a remedy can be prescribed and the avoidance of the otherwise necessary trouble of the patient being obliged to send out to the pharmacy, and when after closing hour being subjected to extra delay as well as to extra expense, it is not difficult to see why the physician should provide himself with a case of remedies for bedside prescribing.

For this purpose it is desirable to select remedies of the greatest efficiency and concentration. As to form, the employment of liquids is not so convenient and cleanly as is the employment of granules and powders.

What is next needed is to provide a system of preparing remedies in the form of powder which shall be practical and comprehensive and be a companion to the dosometric granule.

In the system of decimal triturations of the homœopathic school we have an indication of what is desired. In triturating the remedial agent with an inert powder, preferably sugar of milk, it is an undoubted fact, and most generally admitted by observing practitioners, that the action of the remedy is increased by such process of subdivision —oftimes developing properties which the crude remedy does not possess—and that it is thereby better absorbed and assimilated, and gives better and more prompt results. Now, while the 1X and 2X and in some cases the 3X triturations of the decimal system, which when given in the usual doses of three grains means a dose of either 1-3, 1-33 or 1-333 part of a grain, are allowable and are sensible preparations, it stops at that point, for 4X means a dose of only the 1-3333 part of a grain, which is an amount too minute to warrant further consideration, unless prescribed as a placebo. The centesimal system of trituration, one of the wild vagaries of Hahnemann, does not merit attention.

It will be observed that the principal defect of the decimal system is that the arithmetical progression employed is too rapid. What can be done with the many agents the medicinal dose of which is between the

1-3 and the 1-33 or between the 1-33 and the 1-333 part of a grain? A system of trituration and nomenclature is desired by which this objection will be removed.

I employ a numeral which I have called the medical denominator; hence triturations prepared in accordance with this system can be known as "denominator triturations." In this system, as with the decimal, the medium or general adult dose is uniform, to wit, three grains, which, by the way, can always be accurately measured by means of the three-grain spoon, which at the opposite end is provided with a smaller concavity which when even full contains two grains.

I have triturated one grain of a given alkaloid with a sufficient number of grains of sugar of milk so that three grains shall contain the usual adult dose. As a title after the name I affix a number which is the number of grains containing one grain of the remedy. For example, taking sulphate of morphine, the average dose of which is 1-8 of a grain, I triturate one grain thereof with twenty-three grains of sugar of milk and mark it "Morphiæ Sulphas 24"; of this preparation the number of grains given at a dose is always the numerator over the denominator 24. Thus three grains is 3-24 or 1-8; two grains is 2-24 or 1-12; six grains is 6-24 or 1-4, etc. The remedy is thus sufficiently triturated for accurate dosage and so plainly marked, that, while unintelligible to the layman, it reads in the clearest possible manner to the initiated. The numeral is written or printed in as large type as is the name of the remedy and is on a line therewith, so there is no second line with a large fraction in very small figures to decipher. One soon learns to associate the numeral with the name as a part thereof, and further to classify together those remedies which require the same denominator.

The denominators which I find are the best to employ are 3, 6, 9, 12, 18, 24, 30, 36, 48, 60, 75, 90, 120, 150, 180, 240, 300, 360, 450, 600, 750, and 900; 10 and 100 may also be employed, they being the equivalents respectively of 1X and 2X of the decimal system, and may be substituted one for the other as desired. I do not think that the benefits of trituration are secured when less than two parts of the inert powder are combined with one part of the remedial agent. This system of trituration need not be limited to the alkaloids, but may be employed with the resinoids, glucocides, oleo-resins, or with other agents, as arsenious acid or any other remedy which is employed in small doses. In like manner those popular preparations, the tablet triturates, can be marked with the denominator number and divided in three-grain tablets. If desired, they can be reduced to powder and the required dose measured with reasonable precision, which would also permit of the employment of tablets which have heen accidentally broken. This system of trituration and marking remedies will be found to be both convenient and practical by physicians who care to do any office dispensing. And the specialist may also find it of value in the preparation of powders for topical application, in which case such other inert powder as desired can be substituted for the sugar of milk.

There has within the past year been put upon the market a class of remedies advertised as "alkatrites," which are similar to the preparations which I suggest, inasmuch as they are triturations which it is intended shall be given in three-grain doses, and in each case there is marked on the vial, in small figures, the quantity of the alkaloid which each three grains contains. It can easily be seen that it would prove at least annoying when the dose is increased or diminished to tell easily and quickly the exact amount being given. These remedies are triturated with an inert coloring matter, which is not a bad idea, as it is a proof of thorough trituration.

Dr. Robert Tilley: I supposed from the title of the paper that the doctor was going to talk about a peculiar method of trituration. With reference to his suggestion about the desirability of employing a pocket case; I suppose we all appreciate that—I do, at any rate. As to the use of the tablet triturations, I have been using them for years past. As to eligible preparations, I do not want any more eligible preparations than Fraser's, which give me all that I at present desire: those appearing in the elegant little tablets, practically without taste for the most part, and furnished with the most accurate dosing. I have tested them myself repeatedly. The materials are all of the very best. For instance, referring to the proto-iodide of mercury, commonly and erroneously called the green iodide of mercury, there is no better preparation of proto-iodide of mercury on the market than that which Fraser supplies. It is true he obtains the proto-iodide of mercury from McKesson & Robbins, who have given special study to the proto-iodide of mercury. I have observed and demonstrated that in the large part of the proto-iodide pills on the market, the peculiar green and blackish color, from which the preparation has been called the green iodide, is due to a certain amount of red iodide, a certain amount of metallic mercury, and a certain amount of black oxide, associated of course with the yellow iodide, and these constituents together form that muddy green from which the trituration is called the green iodide. I should think the preparation could be made very much more acceptable by the manufacturer than the physician can make it for himself. The amount of machinery that is used by those who triturate these preparations efficiently is quite considerable, and I am satisfied that any one examining the subject would not suppose that he, in his own office, could triturate as well.

Dr. C. D. WESTCOTT: For a year or more I have been using the triturated alkaloids, as the doctor suggests, in a pocket case, and have had abundant proof of the greater efficiency of the alkaloids over fluid preparations as ordinarily obtained. For instance, in my case I always carry a triturate of aconita or aconitine, the alkaloid of aconite, and have found that by dissolving a certain number of tablets in a certain quantity of water (20 to 30 teasponfuls), I can regulate the amount of medication I desire to get, and obtain a more uniform effect than by the use of any fluid preparation.

Professor G. C. PAOLI said he could not favor the plan of carrying medicines in a satchel so long as he had reliable druggists to depend upon. He believed the fluid extracts made by scientific druggists in Chicago to be pure and to contain the proper medicinal strength. He did not favor granules, for he had known them to pass through the intestinal tract undissolved. He believed the old way to be the best, although he admits the granules are more inviting in appearance. But he cannot yet conscientiously use them.

The PRESIDENT: I was going to ask how the doctor would get along with the different cathartics; there are so few of them from which you could get a valuable alkaloid, and it is so very desirable to use that class of medicines.

Dr. EDWIN PYNCHON: In answer to that question, I will say that there are four or five cathartics from which we can get an alkaloid or glucocide, to wit, aloes, podophyllum, colocynth, leptandra, and jalap; these fill the bill very well. Of course this class of triturations cannot be used for all remedies. In the same way the metric granules are only suitable for those remedies given in a very concentrated form. Fluid extracts are only for remedies which are of vegetable nature, and soluble.

In reply to Dr. Tilley, I will state that it was not my intention that each physician should prepare his own triturations. The phraseology I should have used was that I had them prepared. If there were a demand for medicines prepared in the form of powder there would be plenty of firms who would be glad to so prepare them. The idea of prescribing medicine in powdered form is general. The old Dover's powder is a common prescription, and it certainly ought to be as easy to prescribe a remedy in the nicer form. Alkatrites are being made in large quantities, which indicates that there is a field for such remedies.

As to Dr. Paoli's remarks; I do not wish him to understand that I depend entirely upon a pocket case. In fact I do not depend one-third of the time upon a pocket case, but write prescriptions frequently. As to the idea of alkaloids not being dissolved, my impression is that alkaloids are very soluble, and it is owing to their extreme solubility that they are as powerful as they are. If alkaloids are not soluble, prescribing them in the form of dosimetric granules ought to be more dangerous than prescribing them in the form of powder. As to the idea of following old ways; of course many are in the habit of so doing, and we cannot persuade them to do otherwise. I like the old ways when they are good, but prefer the new ways when they are better.

